# How to select interventions for promoting physical activity in schools? Combining preferences of stakeholders and scientists

**DOI:** 10.1186/s12966-023-01452-y

**Published:** 2023-04-25

**Authors:** Mirko Brandes, Berit Brandes, Louisa Sell, Jennifer M. Sacheck, Mai Chinapaw, David R. Lubans, Alexander Woll, Jasper Schipperijn, Russell Jago, Heide Busse

**Affiliations:** 1 Department of Prevention and Evaluation, Leibniz Institute for Prevention Research and Epidemiology - BIPS, Achterstraße 30, 28359 Bremen, Germany; 2 Department of Exercise and Nutrition Sciences, Milken Institute School of Public Health, The George Washington University, Washington, D.C. USA; 3 Amsterdam UMC, Location Vrije Universiteit Amsterdam, Public and Occupational Health, Amsterdam Public Health Research Institute, Amsterdam, The Netherlands; 4 Priority Research Centre for Physical Activity and Nutrition, University of Newcastle, Newcastle, Australia; 5 Institute of Sports Science, Karlsruhe Institute of Technology, Karlsruhe, Germany; 6 Department of Sports Science and Clinical Biomechanics, University of Southern Denmark, Odense, Denmark; 7 Centre for Exercise, Nutrition & Health Sciences, School for Policy Studies, University of Bristol, Bristol, UK; 8 Population Health Sciences, Bristol Medical School, University of Bristol, Bristol, UK

**Keywords:** Physical activity intervention, Implementation, Longevity, Costs, Efficacy, Reach, Feasibility

## Abstract

**Background:**

The failure to scale-up and implement physical activity (PA) interventions in real world contexts, which were previously successful under controlled conditions, may be attributed to the different criteria of stakeholders and scientists in the selection process of available interventions. Therefore, the aim of our study was to investigate and compare the criteria applied by local stakeholders and scientists for selecting amongst suitable school-based PA interventions for implementation.

**Methods:**

We conducted a three-round repeated survey Delphi study with local stakeholders (*n* = 7; Bremen, Germany) and international scientific PA experts (*n* = 6). Independently for both panels, two rounds were utilized to develop a list of criteria and the definitions of criteria, followed by a prioritization of the criteria in the third round. For each panel, a narrative analysis was used to rank-order unique criteria, list the number of scorers for the unique criteria and synthesize criteria into overarching categories.

**Results:**

The stakeholders developed a list of 53 unique criteria, synthesized into 11 categories with top-ranked criteria being ‘free of costs’, ‘longevity’ and ‘integration into everyday school life’. The scientists listed 35 unique criteria, synthesized into 7 categories with the top-ranked criteria being ‘efficacy’, ‘potential for reach’ and ‘feasibility’. The top ranked unique criteria in the stakeholder panel were distributed over many categories, whereas four out of the top six criteria in the scientist panel were related to ‘evidence’.

**Conclusions:**

Although stakeholders and scientists identified similar criteria, major differences were disclosed in the prioritization of the criteria. We recommend an early collaboration of stakeholders and scientists in the design, implementation, and evaluation of PA interventions.

**Supplementary Information:**

The online version contains supplementary material available at 10.1186/s12966-023-01452-y.

## Background

Physical inactivity is one of the four leading global health risks [1] for disease burden and a reduction in life expectancy [2]. Substantial health benefits are well documented for children who are more active and engage in higher volumes and intensities of physical activity (PA) [3]. As a physically active lifestyle starts to develop in early childhood and tracks into adulthood, the promotion of PA during childhood is of fundamental importance for current and future health status [4]. Schools have been highlighted as key settings for health promotion as children spend many of their waking hours at or in school [5]. A multitude of school-based PA interventions have been developed, evaluated and implemented [6–10]. Whilst these interventions share the same overall goal, to positively impact children’s PA levels, they differ vastly in their approaches, foci and design [11, 12]. Opportunities to promote PA via school might encompass activities such as active breaks during and between school lessons, physical education sessions, after-school programs or active travel to school initiatives [13]. Additionally, in line with a ‘whole-school’ approach, interventions might encompass a combination of components [14].

A recent scoping review identified more than 170 interventions that were reported on in the scientific literature between 2010 and 2019 targeting PA promotion in primary school settings [11]. Whilst the size, sustainability, and equity of effectiveness of school-based interventions to impact on young people’s PA is still debated [9, 10, 15], it is evident that enormous efforts have been undertaken in developing and evaluating school-based interventions to promote PA amongst children and young people. However, the crucial question remains as to which interventions work in the ‘real world’, independent of scientific investigation and short-term funding resources, and as to which interventions have the potential to be successfully implemented and sustained long-term.

Poor implementation within the school setting may explain the drop in effectiveness that is often observed as interventions progress from the early stages of implementation (high levels of research support) to later stages where there is reduced or no researcher support [16]. During these later stages of scale-up and implementation, the implementation is likely driven predominantly by stakeholders, such as school staff, sports club officials or representatives of local administrations. Current research shows that stakeholders often implement changes to the intervention during the scale-up process, in particular if the interventions have to be adapted further (e.g., changing the mode of delivery), which may result in loss of intervention effectiveness [17]. Additionally, changing the delivery agent of an intervention is also associated with a potential loss of efficacy/effectiveness [15]. A review on the sustainability of PA promoting school-based interventions in real-world settings showed that only a limited number of interventions were successful when delivered under real-world conditions or at scale [18]. Many different factors may lead to changes in intervention components or implementation strategies, such as the need to adjust the intervention to the given local context, spatial conditions, school timetable and curriculum, or the mode of delivery. Also, a different sense of what is prioritized by key stakeholders might lead to changes in the intervention. Although traditionally most interventions are developed and evaluated by researchers in relatively controlled conditions, the successful implementation and scale-up is dependent on how school-leaders, teachers and other stakeholders perceive the intervention. It is therefore advisable to address the requirements for a successful physical activity intervention from both scientists’ and stakeholders’ perspectives early on. Therefore, the aim of this study was to investigate and compare the consistencies and discrepancies in criteria of international scientists and local stakeholders for selecting interventions for the promotion of PA in schools.

## Methods

### Study design

We designed and conducted a three-round Delphi study amongst two panels, of community stakeholders and scientists with expertise in PA interventions. Our Delphi study was part of the ACTIvity PROmotion via Schools (ACTIPROS) research project, which aims to set up a toolbox including evidence-based interventions to promote PA in primary school children aged 6 to 10 years. The Bremen University ethics committee approved the study (ref: 2022–08).

### Participants

Two Delphi panels were established to take part in the study, including a local (City of Bremen, Germany) stakeholder panel and an international scientist panel. Stakeholders (*n* = 7) were recruited from the advisory board of the ACTIPROS project, and included one representative of local pediatricians, primary school headmasters, the Bremen State Sports Association, the Bremen Health Association, the State Institute for Schools, a local sports club and the Authority for Children and Education. Scientists (*n* = 6) were recruited through existing contacts and from the reference list of the scoping review on PA interventions in primary schools, conducted in the ACTIPROS project [19]. Only after the Delphi study was completed, local stakeholders and scientists involved in the panel were invited to contribute as co-authors to this paper. All scientists involved accepted this invitation and contributed as co-authors (JS, MCAP, DL, AW, JS and RS).

### Procedures

Participants were approached via email by the lead author (MB), provided with information about the Delphi study and invited to participate. The study was conducted from October 2020 until January 2021.

The overall aim of this Delphi study was to develop and rank criteria which can be applied to assess a large set of interventions retrieved from a previously performed scoping review [11]. Surveys were conducted in a blinded manner. Surveys for both Delphi panels were structured identically and followed the same Delphi methodology of a repeated survey with relevant individuals, as described by McMillan and colleagues [20]. In contrast to the most common aim of Delphi studies, which is to achieve a consensus, our intention was to aggregate criteria to an open question or task (round 1 and 2), and to add a subsequent prioritization (round 3) [21]. All materials provided to the participants (e.g. instructions) were pre-tested by three staff members of the project team (MB, BB, LS). The Delphi study was conducted in English amongst the scientist panel and in German amongst stakeholder panel. Initially, all documentation from the stakeholder panel was conducted in German, but then translated to English independently by two members of the study team. Translations were discussed to derive one final translation. All correspondence was done electronically. The lead author (MB) was available to be contacted at any time in case questions arose. Participants were given two weeks to respond to each round. At no time in the study did the research team add criteria to either panel, enforced criteria or took part in the ranking of criteria.

#### Round 1: Open round

In round 1, information was provided about the background of the ACTIPROS study and toolbox idea of interventions in order to explain the rationale for the Delphi study. Participants were asked *‘Imagine that you have more than 160 PA promoting interventions to choose from’* followed by asking *‘What criteria would you use to decide which of these interventions should be included in the toolbox?’* For this first round, participants were instructed to enter criteria into a table and to define each criterion with 1–2 sentences. Participants were informed that they could enter as many criteria as they liked, that the order of the criteria was irrelevant, and that at least six criteria had to be noted.

Participants’ responses were merged into one separate list for stakeholders and scientists. One member of the project team reviewed the complete list, merged identical criteria and their associated definitions into one criterion and associated definition. A second team member reviewed this process. Changes to the wording were kept as minimal as possible, and care was taken to ensure that the meaning of criteria and definitions were not changed. In cases of disagreement between team members, criteria and definitions were not merged. All changes were done in track changes mode, so that participants were able to track all modifications in round 2. Due to the high number of responses in round 1, criteria were grouped into overarching categories for providing a better orientation for each group of participants. Categories were developed and cross-checked by two members of the project team.

#### Round 2: Reflection and extension

In Round 2, the complete list of assembled criteria for each Delphi panel was forwarded to the participants along with the following statement: ‘*Identify your responses from the attached list and check if they were reproduced adequately. For this purpose, we also attached your individual responses from round one.*’ In addition, participants were instructed: ‘*Reflect on your responses from round one. If you want to, you can now add up to three more criteria for the selection of interventions into the toolbox.*’

To complete round 2, members of the study team added the newly mentioned criteria to the list and integrated any modifications made by participants. Subsequently, all remaining modifications were accepted, and the two resulting lists were cleaned from track changes. The final lists each consisted of a two-column table, one column for the criteria and one for the associated definition.

#### Round 3: Prioritization

For the final round, participants were requested to rank the criteria by assigning a given number of points to the criteria. Participants were instructed to review the final list of criteria and to please ‘*rank the criteria by assigning exactly X points in total to the criteria according to your preferences*’. The number of points was not set a priori. Instead, to allow for assigning at least one point to each criteria, the number of points was defined after finalizing the list after round 2. Because it was likely that stakeholders and scientists accumulate a different number of criteria after round 2, the process allowed for a different number of points for stakeholders and scientists. The absolute number of points was only used to rank order the criteria in the stakeholders and scientists group independently.

The number of points was X = 50 in the scientist group, with a final list of 35 criteria obtained in round 2 in the scientists panel, and X = 100 points in the local stakeholder group, with a final list of 53 criteria obtained in round 2 in the stakeholders panel (see Results). The number of points available to be assigned to criteria was specified by the investigators and was guided by the total number of criteria of the local stakeholders or scientists list, respectively. Participants were free to assign any number of points to each criterion, which meant that all points could theoretically be assigned to a single criterion or that points could be distributed equally across criteria. The criteria were consequently ranked according to their total number of points.

### Analyses

A narrative analysis was performed whereby points assigned to each criterion in round 3 were summed up and the criteria were sorted by the number of points received. The number of scorers was also tracked for each criterion, which included how many stakeholders or scientists gave points to the unique criterion, irrespective on how many points were given. The goal of the analyses was to identify the most important criteria for stakeholders and scientists, respectively, and to compare the ranking of criteria between both groups. MS Excel was utilized for data processing.

## Results

Thirteen participants (seven stakeholders, six scientists) took part in the study. All participants successfully completed the tasks from round 1 to 3. Only in one case did a participant contact the lead investigator to clarify a task.

The local stakeholder panel reported 67 criteria in round 1, which were merged into an initial list containing 44 unique criteria. The following overarching eleven categories were specified: resources (*n* = 5 items); sustainability (*n* = 1); integration (*n* = 3); physical literacy (*n* = 11); staff, support and networking (*n* = 9); parents (*n* = 4); acceptance and emotions (*n* = 4); acceptance and participation (*n* = 5); evidence (*n* = 3); other (*n* = 8, all other criteria that could not be assigned to the previous categories). In round 2, the stakeholders added 9 more criteria to the list, resulting in a final list of 53 criteria for the ranking in round 3.

The scientist panel reported 45 criteria in round 1, which were merged into a list of 31 unique criteria. Criteria corresponded to the following seven categories: evidence (*n* = 9), resources (*n* = 4), adaptability (*n* = 7), acceptance and participation (*n* = 4), feasibility (*n* = 3), acceptance and emotions (*n* = 3), and other (*n* = 5). In round 2, the scientists added four additional criteria to the list, resulting in a final list of 35 criteria, which were ranked in round 3.

The final lists of criteria, including their definitions, the number of scorers per criterion and total number of points per criterion, are available for local stakeholders (Additional file 1) and scientists (Additional file 2). Table 1 presents the top 13 ranked criteria by the stakeholders and scientists panel, respectively.

**Table 1:** Top 13 ranked criteria of local stakeholders and scientists. Please refer to Additional files 1 and 2 for criterion definitions

	**Local stakeholders**			**Scientists**			
**Rank**	**Category**	**Criterion**	**Points**	**Rank**	**Category**	**Criterion**	**Points**
1	Resources	Free of costs	47	1	Evidence	Efficacy	35
2	Sustainability	Longevity	39	2	Evidence	Potential for reach	25
3	Integration	Integration into everyday school life	38	3	Feasibility	Feasibility	20
3	Physical literacy	Teampromotion (cooperative and competition)	38	3	Acceptance and emotions	Child likeability/ acceptability	20
5	Staff, support, networking	Mentoring-model, training of school sports assistants (peers)	37	5	Evidence	Ease of implementation	15
6	Parents	Relevance to everyday life—transferability	32	5	Evidence	Sustainability	15
7	Staff, support, networking	Organisation	31	5	Acceptance and participation	Participation/ stakeholder involvement (children, school staff, parents, policymaker)	15
8	Staff, support, networking	Qualification	29	5	Other	Systems-approach	15
9	Physical literacy	Self-efficacy / learning success	26	9	Evidence	Positive implementation trial	10
10	Staff, support, networking	Feasibility—support	22	9	Resources	Intervention cost	10
10	Acceptence and emotions	Attractiveness	22	9	Adaptability	Tailoring	10
10	Other	Complexity	22	9	Adaptability	Reducing inequalities	10
13	Resources	Funding	20	9	Acceptance and emotions	Enjoyment	10

Rank: The criteria were rank-ordered by their number of points. Points: sum of points assigned to the criterion by stakeholders and scientists, respectively

In the stakeholder panel, the criterion ‘free of costs’ was ranked first, with a sum of 47 out of 700 points received from 5 out of 7 stakeholders (‘scorers’). Hereafter, the criteria ‘longevity’ (39/700; 5 scorers), ‘integration into everyday school life’ (38/700; 5 scorers), ‘team development (cooperation and competition)’ (38/700; 4 scorers) and ‘mentoring-model, training of school sports assistants (peers)’ (37/700; 4 scorers) were ranked highest. In contrast, ‘efficacy’ scored best in the scientist panel, with 35 out of 300 points (3 out of 5 scorers). On the following ranks, ‘potential for reach’ (25/300; 3 scorers), ‘feasibility’ (20/300; 2 scorers) and ‘child likeability/acceptability’ (20/300; 2 scorers) scored highest.

Based on the agreements between stakeholders and scientists as well as considering priorities of both groups in our study, we propose a set of key criteria to the selection process of PA interventions in primary school children which is outlined in Table 2.

**Table 2:** Set of 7 key criteria to select PA interventions. Origin represents ranking of criteria by scientists (Sc) and stakeholders (St)

Criterion	Definition	Origin
Costs	Costs to deliver the intervention, including additional resources, equipment, and maintenance, minus costs covered by external funding	Sc: 9^th^ St: 1^st^, 13^th^
Sustainability	Likelihood of the intervention to be continued after implementation project has expired, without external support	Sc: 5^th^ St: 2^nd^
Feasibility	Fitting/Adoption possibilities to spatial–temporal school routines and conditions	Sc: 3^rd^, St: 3^rd^
Potential for reach	Program reaches a wide variety of children in terms of age, sex, race/ethnicity, fitness level, socioeconomic status, etc	Sc: 2^nd^ St: 21^st^
Efficacy	Effects on children´s PA and health benefits are scientifically proven	Sc: 1^st^ St: 18^th^
Acceptability	Acceptability is ensured by enjoyment, fun and likeability, reported by the children	Sc: 3^rd^, 9^th^ St: 10^th^
Social development	The intervention offers opportunities for social development, such as cooperation, competition	St: 3^rd^

## Discussion

We conducted a three-stage Delphi survey to investigate and compare key criteria for selecting interventions for the promotion of PA in primary school children from the perspectives of local stakeholders as well as a team of international PA scientists. The top six criteria in the stakeholders group originated from six different categories. In contrast, the category ‘evidence’ dominated in the scientists’ group by representing four out of the top six criteria. Although being expressed in different words, many criteria overlapped between stakeholders and scientists. When considering only the top ten criteria of each group, three criteria were identified that were named by both stakeholders and scientists, namely costs, sustainability and feasibility of the intervention. ‘Free of costs’ was ranked first by the stakeholders, and ‘intervention costs’ was ranked ninth by the scientists. The criterion ‘longevity’ ranked second by the stakeholders and ‘sustainability’ ranked fifth by the scientists. Additionally, both groups ranked the feasibility of the interventions, as defined as the ease of integration of the intervention components into school routines, as high (ranked third in both groups). The first evidence-related criterion in the stakeholders’ group was ‘empiricism’ ranked 18^th^.

### Unique criteria

Regarding the best-rated unique criteria, ‘free of costs’ was the highest ranked criterion amongst local stakeholders, whereas the related criterion ‘intervention costs’ by the scientists ranked only 9^th^. In contrast, the scientists’ top-ranked criterion ‘efficacy’ was only 18^th^ in the stakeholders ranking. We assume that this difference is highly driven by the different viewpoints of stakeholders and scientists. Stakeholders may argue that they do not want to pay extra for the interventions, such as staff deployment and materials. In contrast, scientists may argue that no intervention is completely free of costs (someone has to contribute time and resources for any intervention). Instead, the return of investment (ROI) is crucial, which is incorporating the efficacy of an intervention: given fixed costs for an intervention, the ROI increases with higher efficacy. For example, comprehensive school health programmes were found to be cost-effective per quality-adjusted life year gained for up to CA$682 [22] and for increasing PA levels in secondary schools [23]. Therefore, scientists relate the costs associated with an intervention to the ROI, whereas stakeholders presumably (have to) focus on the short-term costs only, maybe because the ROI does not pay back into the schools´ budget. Consequently, the long-term health benefits of children, which are highly relevant for estimating the ROI, may be less top of mind for stakeholders.

Stakeholders highly ranked ‘longevity’ of the intervention and the ‘integration into everyday school life’, while scientists highly ranked ‘potential for reach’ and ‘feasibility’. In the case of ‘integration into everyday school life’ and ‘feasibility’, both definitions are similar and it is possible that stakeholders and scientists were referring to the same thing. The same holds true for the sustainability of interventions, what was named ‘longevity’ by the stakeholders. For criteria related to the reach of an intervention, the stakeholders named several unique criteria, which could potentially be summarized to a broader criterion called ‘reach’. Despite the criterion ‘reach’, which obtained 15 points in total, other criteria such as ‘gender sensitivity’, ‘intercultural movement programs’ and ‘group size’ add the stakeholders’ view on the reach of an intervention, highlighting the stakeholders attention paid to the representativeness of the sample, which stands in relation to the potential reach of an intervention. However, the scientists rated ‘reach’ as much more important compared to the stakeholders. Further noteworthy findings included criteria related to the social development of children were not mentioned at all by the scientists, and that the child likeability/attractiveness of the interventions scored only low in the stakeholders view.

### Categories

The stakeholders scored well across many categories, reflected by eight different categories in the top 13 (see Table 1), demonstrating the broader view on what is important to select an intervention for the school setting. In contrast, the category ‘evidence’ dominated the top 13 of the scientists and was represented five times (four times in the top 5, see Table 1). This might not be surprising, given the focus of researchers to ensure internal validity, e.g. to control conditions for a scientifically sound efficacy trial. However, this might feed the suspicion of a too tight view of what is relevant for running successful interventions in schools, as there must be flexibility at large scale interventions, highlighting the need for external validity. Depending on the type of trial along the scale-up continuum, the importance of internal and external validity will vary. At least in part, the voltage drop that occurs as interventions progress from efficacy trials to dissemination may result from the focus on internal validity in smaller scale trials and may be a barrier to achieving effects at scale [15].

### Synthesis

Comparing our synthesized results (see Table 2) directly to other studies is difficult due to the low number of specific studies in this area. Tibbitts et al. analysed stakeholders’ perspectives on why some individual-level approaches of PA promotion failed in UK schools in recent years [24]. Six out of the seven most important stakeholders’ statements in this study are similar to our proposed key criteria (Table 2). The only missing criterion not mentioned in the study by Tibbitts and colleagues was ‘costs’ [24] even though our study found it to be ranked high amongst the stakeholders and scientists. With respect to scientists, our proposed set of 7 key criteria (except ‘social development’) nicely maps to the implementation outcomes and determinants suggested for the evaluation of implementation and scale-up of PA and behavioural nutrition interventions as suggested by McKay and colleagues, although their findings are based on research including only scientists [25]. In order to facilitate the selection of promising and suitable interventions for stakeholders and practitioners, we suggest that researchers address the key criteria mentioned above, potentially in combination with the template for intervention description and replication (TIDieR) checklist [26], and make use of online supplements or other digital resources to provide more detailed information about the intervention. Given the importance of the ‘intervention-context’ fit, we further recommend researchers to a) involve stakeholders in the decision-making as early as possible, and b) report more detail upon the context and implementation of a given intervention, e.g. by utilizing the PRACTical planning for Implementation and Scale-up (PRACTIS) guide [27]. This will help others to establish the prerequisites and requirements that made a given intervention ‘work’ within a certain setting and context.

The findings of our study can also be used to support national or international approaches of PA promotion, e.g. putting the Global Action Plan on Physical Activity 2018–2030 (GAPPA) of the WHO into practice [28]. Recently, the International Society for Physical Activity and Health (ISPAH) highlighted the whole-of-school programmes as one of the ‘Eight Investments That Work for Physical Activity’ [29]. Convincing stakeholders is considered one of the key components of successful promotion of PA by the authors [29], demonstrating the urgent need to bring stakeholders’ and scientists’ views together.

Co-developing and co-designing interventions together with stakeholders (including the school children themselves) is a promising approach to overcome the ‘real-world implementation’ barriers right from the start. It is assumed that these so-called ‘authentic partnerships’, including but not limited to teachers, children, executive staff, policy makers, educational and health care professionals are key to successful implementation of PA interventions at large scale (for further details, please refer to Kennedy et al., 2020 [30]). Additionally, close stakeholder engagement throughout the evaluation and the possible adaptation of an intervention can further improve the acceptability and feasibility of interventions. Whilst some studies have involved teachers in the implementation, adoption and sustainability of school-based interventions [31], future studies might also involve important others, such as parents, children and educational authorities, when developing, implementing and evaluating interventions.

#### Strengths and limitations

To our knowledge, this is the first Delphi study combining scientists’ and stakeholders’ views on which criteria are most important for selecting school-based interventions to promote PA. It adds on the findings of Daly-Smith and colleagues, who successfully co-developed the Creating Active Schools Framework with practitioners, policymakers and researchers in the UK [32]. Importantly, our study included international PA intervention experts from different continents in conjunction with local stakeholders from a German community. All participants completed all stages of the study. Furthermore, our study design allowed for iteration and controlled feedback, meaning that participants had the opportunity to adapt their opinions and to add criteria in subsequent rounds. This allowed participants to re-think and re-evaluate their views over the duration of the study, instead of providing views only at a single point in time.

However, multiple limitations also need to be considered. A limitation is that we did not include children in the Delphi process. As most decisions on intervention selection in a specific school is currently taken by adults, we did not incorporate children at this stage. However, ensuring that an intervention is acceptable to the target audience is of course a key aspect for success. It is inevitable to include children in the decision-making process when it comes to the acceptability of a specific intervention. Whilst important for our future study, including local stakeholders from Bremen only might mean that the findings from the stakeholders are not representative for other areas in Germany or elsewhere. It would be interesting to investigate whether stakeholders in other areas share the views of the local stakeholders or differ in their views in future research. Further, we acknowledge the small number of participants included in our study as a limitation. Whilst some Delphi studies include ‘discussion’ phases amongst participants, we felt that this was not appropriate for this study as we were interested in keeping the two panels separate, investigating and comparing both panels’ view. We thus decided not to combine the panels nor introduce phases of discussion amongst scientists and stakeholders, not only because of language differences, but also because we did not want stakeholders to be influenced by scientists and vice versa.

## Conclusions

Stakeholders and scientists demonstrated a fair overlap of criteria when selecting among school-based PA interventions, however, they substantially differed in their characterization and ranking which limited the agreement between both groups. In order to take into account both sets of criteria, we suggest a) to ensure a sound collaboration of stakeholders and scientists in design, implementation and evaluation of PA interventions, and b) to advance the mutual understanding and agreement between both groups by developing a harmonized position. Given our current findings, we suggest to build the communication process between stakeholders and scientists on the set of seven key criteria (costs, sustainability, feasibility, potential for reach, efficacy, acceptability and social development). A better understanding and agreement provided, we assume positive impact at least on the implementation, effectiveness and sustainability of physical activity interventions.

## Supplementary Information

**Additional file 1. **Final list of criteria and definitions by the local stakeholders.

**Additional file 2. **Final list of criteria and definitions by scientists.

## Data Availability

The datasets used and/or analysed during the current study are available from the corresponding author on reasonable request.

## Abbreviations

PA: Physical activity
ACTIPROS: ACTIvity PROmotion via Schools
Sc: Scientists
St: Stakeholder
ROI: Return of investment
TIDieR: Template for intervention description and replication
PRACTIS: PRACTical planning for Implementation and Scale-up guide
GAPPA: Global Action Plan on Physical Activity
ISPAH: International Society for Physical Activity and Health
