# Supplementary material for: How to select interventions for promoting physical activity in schools? Combining preferences of stakeholders and scientists
Source: Int J Behav Nutr Phys Act. 2023 Apr 25;20:48. doi: 10.1186/s12966-023-01452-y (PMC10127415; doi:10.1186/s12966-023-01452-y)
Supplement: Supplementary file 1 — Additional file 1. Final list of criteria and definitions by the local stakeholders. [file 12966_2023_1452_MOESM1_ESM.docx]

**Additional file 1. Final list of criteria and definitions by the local stakeholders.**

| **Stakeholder** | | | | |
| --- | --- | --- | --- | --- |
| **Category** | **Criterion** | **Points** | **# Scorer** | **Description** |
| Resources | free of costs | 47 | 5 | Intervention is free of charge for the receiver |
| Resources | funding | 20 | 1 | Funding opportunities for the intervention are available (foundations, etc.) |
| Resources | feasibility - effort and costs | 18 | 3 | Intervention is easily implemented with limited resource and maintenance costs. |
| Resources | consider rooms | 13 | 2 | Many schools lack of suitable spaces. Gyms are in some cases fully occupied or not usable. Therefore interventions should be made possible outside of school spaces and gyms. |
| Resources | feasibility material requirements | 16 | 3 | Special- or daily materials, storage, distribution |
| Staff, support, networking | personnel effort of the school | 2 | 1 | Intervention does not require much time of school staff to be introduced and repeated. |
| Staff, support, networking | quality of support | 11 | 2 | Does it need the support of a network around a school, to carry out an intervention? If yes, in which quantity and quality. |
| Staff, support, networking | qualification | 29 | 4 | Intervention can be run with limited previous knowledge and does not require specific qualifications |
| Staff, support, networking | Mentoring-model, training of school sports assitants (peers) | 37 | 4 | Intervention includes peer-to-peer approach to physical activity |
| Staff, support, networking | feasibility - support | 22 | 3 | Intervention can be conducted independently following training; external support |
| Staff, support, networking | level of independence | 16 | 3 | Intervention can be run independently by students |
| Staff, support, networking | organisation | 31 | 2 | Intervention receives assistance from external partners as school already have a high burden of tasks. |
| Staff, support, networking | partner networking | 18 | 2 | Encouragement for networking, for example in community; Partners necessary/ helpful. |
| Staff, support, networking | lobbying / public relations | 4 | 1 | Intervention highlights added value that can be gained for schools through physical activity, such as in the combination of being active and learning successfully. |
| Parents | acceptance by parents | 11 | 2 | Some interventions require a higher investment of time by parents or legal guardians and therefore don't receive a lot of acceptance. In view of time, the interventions should be fitted in the daily routine/structure of families. |
| Parents | parents | 11 | 3 | Inclusion of the parents to continue physical activities within the private homes. |
| Parents | relevance to every day life - transferability | 32 | 4 | Relation to school or extracurricular daily routine usable, independently realisable by pupils and families. |
| Parents | integration of families | 12 | 2 | Intervention involves the whole family. Families should be invited to participate in related activities as to support their children and to integrate the components into family life. |
| Integration | integration into everyday school life | 38 | 5 | Intervention should be easily integrated into the daily school routine. |
| Integration | character / type of the intervention | 2 | 1 | Does an intervention aim at PE, at elective subjects or workgroups, small or big breaks, exercise breaks during lessons, everyday actions or leisure behaviour? |
| Integration | rhythmization | 12 | 2 | Complement education with physical activity, apprehend physical activity as a part of everyday school life and integrate it in the daily rhythm. |
| Physical literacy | sphere of action | 8 | 2 | Intervention aims to address multiple aspects of physical activity promotion (e.g. personal development, promotion of coordination, etc.). The more aspects can be addressed by the intervention, the better. |
| Physical literacy | promotion of coordination skills | 6 | 2 | Intervention should promote coordination skills |
| Physical literacy | warming up / rhythmization | 0 | 0 | Small, easy and diverting games that for example contribute in small breaks to the rhythmification of lessons by physical activity. |
| Physical literacy | condition: strenght / speed / endurance | 17 | 3 | Intervention should consist of activities relevant to the age of participants. Activities should support the development of participants and challenge ‘borderline experiences’ in handling their own bodies. |
| Physical literacy | speed of action / complex decisions / complex decision / concentration | 1 | 1 | Intervention should consist of movement-oriented reaction games that require a quick action-reaction chain to promote students' cognitive development |
| Physical literacy | tactical grasp | 0 | 0 | Intervention should consist of more complex movements that provoke tactical grasp and decision-making processes |
| Physical literacy | teampromotion (cooperative and competition) | 38 | 4 | Intervention should promote forms of play that involves competitive elements, as dealing with loss and win can promote social behaviours. |
| Physical literacy | self-efficacy / learning success | 26 | 4 | Tasks, that perpetuate and become more complex and are particularly beneficial for self-perception through fast success / improvement. |
| Physical literacy | fun / activation | 3 | 1 | Easy and low-threshold forms of movement or physical activity that particularly by story-telling arouse a desire for physical activity without physical activity itself being the focus. |
| Physical literacy | technology | 1 | 1 | Intervention should cover basic techniques of a range of different sporting activities (throwing, catching, hitting, running,…) |
| Physical literacy | avoid pressure of perform - still promote the willingess to make an effort | 2 | 1 | Intervention should avoid pressure to perform whilst still promoting willingness to put effort into activities |
| Acceptance & Emotions | motivation / volition | 2 | 1 | Intervention should include mentality-promoting forms of movement that stem from intrinsic motivation and that promote volition to achieve a goal. |
| Acceptance & Emotions | motivation / news value | 1 | 1 | Intervention should motivate students, school staff and school to get involved |
| Acceptance & Emotions | attractiveness | 22 | 3 | The intervention has to be appealing on a high scale to be successful. For this, the view of pupils is decisive. |
| Acceptance & Emotions | connection of movement interventions and promotion of health literacy | 3 | 1 | Intervention allows for a better understanding of aspects of movement which can further increase health competencies and health literacy |
| Acceptance & Participation | reach / specificity | 15 | 1 | Intervention is suitable for a broad target audience |
| Acceptance & Participation | group size & supervision key of the interventions | 7 | 2 | How big can a target group be that takes part in the intervention and what is the child care ratio of the intervention? The more children can be reached and efficiently activated, the better. |
| Acceptance & Participation | adaption / variety | 12 | 2 | Intervention can be easily adopted to needs of target population |
| Acceptance & Participation | creation of gender sensitive programmes | 18 | 3 | Intervention is gender-sensitive and should allow for participation by everyone |
| Acceptance & Participation | intercultural movements programmes | 2 | 1 | Intervention is culturally-sensitive and may incorporate different cultural activities |
| Sustainability | Longevity | 39 | 5 | Intervention allows for continuation based end of intervention |
| Evidence | evaluation | 2 | 1 | If possible there should be a short evaluation of the intervention implemented. This can take place by feedback-rounds with the persons responsible (teachers, trainers) and with the children. |
| Evidence | activity / passivity | 1 | 1 | Intervention should require active participation of participants |
| Evidence | effect | 7 | 2 | Effectiveness of intervention can be evaluated and measured |
| Other | complexity | 22 | 3 | Is the intervention easy to mediate or do participants need basic knowledge to better take part in the intervention? The less complexity, the better. |
| Other | health | 11 | 2 | Intervention aligns to current regulations and can be adapted easily (e.g. hygiene concept, etc.) |
| Other | obligatory event | 0 | 0 | Intervention should be mandatory and should entail components that can be selected |
| Other | extent of the intervention | 0 | 0 | Repetitions and time required for intervention per student |
| Other | empiricism | 16 | 3 | Has the intervention already been empirically examined and are effects already empirically proven? The more empiricism present, the higher the acceptance of the intervention. |
| Other | feasibility - accessibility | 1 | 1 | Intervention content should be easily accessible, the use is evident |
| Other | outdoor activities | 7 | 2 | Intervention should be able to be implemented regardless of weather with suitable closing and changing opportunities |
| Other | integration of nature and movement | 8 | 2 | Intervention should entail components that are delivered outside/in the nature to take into account specific environment |

Points: sum of points assigned to the criterion by stakeholders. # Scorer: number of stakeholders who assigned points to the criterion.
