# Supplementary material for: How to select interventions for promoting physical activity in schools? Combining preferences of stakeholders and scientists
Source: Int J Behav Nutr Phys Act. 2023 Apr 25;20:48. doi: 10.1186/s12966-023-01452-y (PMC10127415; doi:10.1186/s12966-023-01452-y)
Supplement: Supplementary file 2 — Additional file 2. Final list of criteria and definitions by scientists. [file 12966_2023_1452_MOESM2_ESM.docx]

**Additional file 2. Final list of criteria and definitions by scientists.**

| **Researcher** | | | | |
| --- | --- | --- | --- | --- |
| **Category** | **Criterion** | **Points** | **# Scorer** | **Description** |
| Evidence | Evidence-based | 5 | 1 | Is the intervention based on scientific evidence, theory or practice-based evidence? |
| Evidence | Potential for reach | 25 | 3 | Programming reaches a wide variety of children in a given contest – by age, sex, race/ethnicity, fitness level, socioeconomic status, etc. |
| Evidence | Efficacy | 35 | 3 | Was the intervention successful in increasing children’s PA? Is the dose of physical activity enough to provide health benefits for students? |
| Evidence | Positive implementation trial | 10 | 1 | Positive results from an efficacy trial are a good start, but positive results from an implementation trial are a much better indication of real-world effects |
| Evidence | Ease of implementation | 15 | 3 | Administrators of the program can be readily and easily trained and in turn, are able to effectively deliver programming to children |
| Evidence | Process evaluation availability | 0 | 0 | Many interventions have been tested in an efficacy trial, with little attention to what a real-world implementation would look like – an intervention with published process evaluation results has at least given implementation some thought |
| Evidence | Description of implementation process | 5 | 1 | A good ‘how-to-manual’ is essential for an intervention, and especially for the intervention process for more complex interventions |
| Evidence | Sustainability | 15 | 3 | Is the intervention likely to be sustained over time without external support from the research team or external providers? |
| Evidence | Potential for sustainability | 5 | 1 | Several other definitions here could fold into potential for sustainability – cost, ease of implementation, reach of programming, acceptability and likeability of programming by children and the implementers (schools administrators, teachers, champions, etc.), and potential to innovate over time |
| Resources | Intervention cost | 10 | 2 | How much will it cost to deliver the intervention? Are additional resources and equipment needed? Who will provide funding to support implementation over time. |
| Resources | Costs | 0 | 0 | Clear project budget of sustainability costs in the project calculations |
| Resources | Resources |  |  | It must provide all of the resources to deliver |
| Resources | Training/support | 1 | 1 | How much training will be required for teachers to deliver the intervention? Will teachers be willing to complete the training? |
| Adaptability | Compatability | 7 | 2 | Extent to which an intervention fits with the mission, priorities, and values of the school system. |
| Adaptability | Tailoring | 10 | 2 | Is the intervention tailored to the specific physical, social, cultural environment? |
| Adaptability | Adaptability | 6 | 2 | Extent to which the intervention can be adapted, tailored, refined, or reinvented to meet the needs of teachers, students and schools |
| Adaptability | Appropriateness | 5 | 1 | Is the intervention appropriate for schools and are children likely to enjoy participating in the intervention? |
| Adaptability | Possibilities for individualization | 0 | 0 | Individual personal motives/performances should be considered |
| Adaptability | Reducing inequalities | 10 | 1 | Does the intervention contribute to reducing inequalities i.e. are those most in need adequately reached and involved? |
| Adaptability | Connectivity | 0 | 0 | Programming that is offered in a way that complements other pre-existing programming that currently does not exist in the setting in order to offer a broader array of activities and increase the likelihood of children meeting PA guidelines |
| Acceptance and Participation | Acceptance in the leadership | 0 | 0 | Commitment of school leadership with the topic |
| Acceptance and Participation | Participation/ stakeholder involvement (children, school staff, parents, policymaker) | 15 | 2 | Are the relevant stakeholders involved in the development i.e. children, school staff, parents, policymakers etc? |
| Acceptance and Participation | Participation (school leaders, teachers, students) | 0 | 0 | More involvement of the people involved in the intervention leads to more commitment, and with that, a better chance of a long term ‘culture-change’ that can lead to sustainable results |
| Acceptance | Acceptance and participants | 0 | 0 | Written strategic aims in the mission statement of the school, clear responsibilities in the organisational structure of schools |
| Feasibility | Feasibility | 20 | 2 | Can the intervention be delivered feasibly in a school setting? |
| Feasibility | Little barriers | 1 | 1 | Not too much additional organizational effort teacher/pupils |
| Feasibility | Complexity | 0 | 0 | Teachers’ perceptions of the intervention, in terms of its difficulty to understand and use; and the number of different intervention components |
| Acceptance and emotions | Child likeability/ acceptability | 20 | 2 | Children readily engage in programming and find it acceptable – they like it/look forward to it |
| Acceptance and emotions | Fun | 0 | 0 | Is the intervention ‘fun’ for children themselves, however this should be evident based on above mentioned criteria |
| Acceptance and emotions | Enjoyment | 10 | 2 | It must be enjoyable for pupils and delivers |
| Other | Dose of activity delivered | 0 | 0 | Programming provides an adequate dose of physical activity. Dose may include extended movement time at lower intensities (light/moderate) or shorter bouts of more moderate-to-vigorous activity – either minimizing sedentary time. Given current guidelines, a focus on MVPA may be more critical. |
| Other | PA skills building/ physical literacy | 5 | 1 | Programming that considers building competency, confidence and motivation to be physically active across a range of movement skills |
| Other | Combined programs | 0 | 0 | Environmental and behaviour oriented programs/measures |
| Other | Systems-approach | 15 | 2 | Does the intervention involve a systems approach i.e. do changes or actions target the most important parts of the system, their interrelatedness, interactions and dynamics in order to produce sustainable and impactful behavioural change? |
| Other | Dose of PA ‘delivered’ vs. ‘uptake’ | 0 | 0 | Often times programming will offer a set of activities or a time to be active – however, this does not necessarily translate to child uptake/engagement for that set of activities or time. (This is part of participation and reach but needs more careful consideration in terms of the efficiency of the programming) |

Points: sum of points assigned to the criterion by scientists. # Scorer: number of scientists who assigned points to the criterion.
